# ERP measures of math anxiety: how math anxiety affects working memory and mental calculation tasks?

**DOI:** 10.3389/fnbeh.2015.00282

**Published:** 2015-10-26

**Authors:** Manousos A. Klados, Panagiotis Simos, Sifis Micheloyannis, Daniel Margulies, Panagiotis D. Bamidis

**Affiliations:** ^1^Max Planck Research Group for Neuroanatomy and Connectivity, Max Planck Institute for Human Cognitive and Brain SciencesLeipzig, Germany; ^2^Group of Applied and Affective Neuroscience, Lab of Medical Physics, Medical School, Faculty of Health Sciences, Aristotle University of ThessalonikiThessaloniki, Greece; ^3^School of Medicine, University of CreteHerakleion, Greece; ^4^Neurophysiological Research Laboratory (L. Widén), School of Medicine, University of CreteHerakleion, Greece

**Keywords:** mathematical anxiety, mathematical cognition, working memory, ERPs, EEG, mental calculations

## Abstract

There have been several attempts to account for the impact of Mathematical Anxiety (MA) on brain activity with variable results. The present study examines the effects of MA on ERP amplitude during performance of simple arithmetic calculations and working memory tasks. Data were obtained from 32 university students as they solved four types of arithmetic problems (one- and two-digit addition and multiplication) and a working memory task comprised of three levels of difficulty (1, 2, and 3-back task). Compared to the Low-MA group, High-MA individuals demonstrated reduced ERP amplitude at frontocentral (between 180–320 ms) and centroparietal locations (between 380–420 ms). These effects were independent of task difficulty/complexity, individual performance, and general state/trait anxiety levels. Results support the hypothesis that higher levels of self-reported MA are associated with lower cortical activation during the early stages of the processing of numeric stimuli in the context of cognitive tasks.

## Introduction

Mathematics plays a crucial role in everyday life, affecting academic achievement, job decisions and performance (Jones et al., 2012). It is not uncommon, however, to experience math-related anxiety in the form of feelings of tension, apprehension, or fear when confronted with even simple mathematical problems (Ashcraft, 2002). High levels of mathematical anxiety (MA) are strong predictors of future academic choices (Fennema, 1989), yet the actual association between levels of self-reported MA and performance on arithmetic tasks or math achievement is in the small to moderate range (Betz, 1978; Ashcraft and Faust, 1994; Ma, 1999). Perhaps the most influential formulation derives from Attentional Control Theory (ACT; Eysenck et al., 2007) which postulates that anxiety, in general, affects the ability to allocate attentional and cognitive resources to task performance. In the case of arithmetic tasks working memory (chiefly the central executive) appears to hold a key role in the coordination of arithmetic-specific skills and knowledge during task performance (Imbo and Vandierendonck, 2008; Raghubar et al., 2010; Klados et al., 2013), while LeFevre et al. (2005) have aptly documented the role of working memory in mathematical cognition. According to ACT, anxious individuals need to exert greater cognitive effort and make use of more cognitive resources in order to achieve performance standards displayed by persons with low anxiety. The engagement of compensatory cognitive strategies and processes would increase processing (and reaction) time in order to achieve adequate accuracy levels.

Given the significant variability in previous studies of performance correlates of anxiety (e.g., Basten et al., 2012), there have been several attempts to better understand the underlying neural processes that mediate the postulated impact of anxiety. The majority of studies have explored general anxiety effects on various cognitive tasks (including tasks of working memory) with mixed results (e.g., Amodio et al., 2008; Fales et al., 2008; Bishop, 2009; Aarts and Pourtois, 2010; Judah et al., 2013). Relatively few neuroimaging studies have shed light on the impact of MA on brain activation patterns associated with processing of numbers and with the performance of arithmetic tasks. For instance, Lyons and Beilock (2012b) recently showed that high levels of MA in anticipation of arithmetic stimuli in adults were associated with increased activity in regions involved in pain perception, as well as in frontoparietal areas (Lyons and Beilock, 2012a). Moreover, increased activity in the caudate, nucleus accumbens, and hippocampus correlated positively with math performance. Electrophysiological studies that focus on MA are also scarce. Recently, enhanced error-related negativity on a numeric Stroop task (Suárez-Pellicioni et al., 2013) and increased late positivity (P600/P3b) elicited by markedly erroneous solutions to simple addition problems (Suárez-Pellicioni et al., 2013) were reported in persons scoring high on a MA self-report scale.

While these studies have provided initial evidence of changes in neural activity associated with MA, important questions remain unanswered. Pertinent issues that the present research was designed to address include: (a) the specificity of math-related anxiety as a characteristic trait that is distinct from both general trait anxiety and general situational anxiety in impacting brain activity; (b) the spatial and temporal features of math-anxiety related effects on neurophysiological activity during the performance of arithmetic tasks. The former issue was addressed by concurrently obtaining measures of both general and math-related trait anxiety as well as state anxiety during the testing session from all participants. The later issue was addressed by obtaining measures of brain activity with adequate temporal resolution to determine during which time window(s) of event-related potential records anxiety effects would occur during processing of numeric stimuli.

In this framework traditional measures of electrophysiological reactivity (ERP amplitude) were obtained in typically achieving young adults who were grouped on the basis of self-reported, anticipatory math anxiety. In addition, ERPs were collected during performance of a common working memory task (single-digit n-back) in view of the close dependence of arithmetic performance upon working memory capacity (e.g., Raghubar et al., 2010).

The primary goal of the study was to assess systematic variability as a function of the level of trait math-related anxiety reported by participants prior to their engagement in arithmetic tasks. We hypothesized that participants reporting higher levels of trait math anxiety would demonstrate reduced ERP amplitudes prior to response selection (Qi et al., 2014). This finding would be consistent with the notion that high levels of math anxiety can impact the neural functioning critically involved in the early stages of processing of numeric stimuli and in basic math-related cognitive operations. The second goal of the study was to examine if the effects of math anxiety on ERP measures are specific to the performance of arithmetic tasks or also evident during performance of a working memory task employing single-digit numeric stimuli. The third goal of the present study was to assess if ERP effects would reflect individual differences in math anxiety rather than more generalized state or trait anxiety characteristics of the participants.

## Materials and Methods

### Participants

With permission from the Bioethics Committee of the Medical School of Aristotle University of Thessaloniki (in agreement with the Declaration of Helsinki), 1000 university students were administered the Greek adaptation of the nine-item Abbreviated Math Anxiety Scale (AMAS; Hopko et al., 2003). Moreover, they have also signed a written informed consent prior to the experimental procedure. Sixteen students (eight men and eight women) out of 63 who scored in the upper 15th percentile of the sample distribution (≥28 points) and did not meet other exclusionary criteria (non-right handedness, history or referral for diagnosis of neurological or psychiatric disorder—including learning disability) were randomly chosen to form the High Math Anxiety (HMA) Group. From the pool of 68 students who scored in the lower 15th% tile on AMAS (≤14 points) we selected 16 who were individually matched with HMA students on gender and age to form the Low Math Anxiety (LMA) Group (Supplementary Figure 1). The two groups did not differ on age (HMA: mean age = 22.21 ± 2.43 years, LMA: 22.50 ± 2.3 years; *p* = 0.73) or gender distribution (8 men and 8 women in each), and they were all right handed adults. The ratio of students completing science degrees over humanities was 12/4 in the HMA and 15/1 in the LMA groups. Although general math ability was not assessed through standardized achievement tests, the experimental tasks used in the present study involved elementary-school level knowledge and skills so that the two groups consisting of college students should perform at comparable levels.

### Experimental Tasks

All participants were administered a working memory task (N-back with three levels of load/difficulty) and four arithmetic tasks (Single Digit Addition, Double-Digit Addition, Single Digit Multiplication, and Double-Digit Multiplication). In the one-back condition participants pressed one mouse button to indicate that the current stimulus (single digit) was the same with the one immediately preceding it and the other button for a “No” answer. In the two- and three-back conditions, participants were asked to compare the current stimulus with the one presented either two or three positions before, respectively. A total of 40 trials (single digit numbers) were presented in each n-back condition.

Each arithmetic task consisted of 40 trials (problems), except Double-Digit Multiplications involving 20 trials in order to avoid frustration of both groups due to their difficulty, presented in a randomized order across participants. Each stimuli was presented visually and remained on the screen until the participant indicated whether it was correct or not by pressing the right or left mouse button, respectively, while the split of the false answers was small. The correspondence of response keys to type of response was counterbalanced across participants. The order of tasks was also counterbalanced across participants.

### Anxiety Measures

The abbreviated version of AMAS consists of nine items representing common situations faced by students (e.g., “Thinking about an upcoming math test one day before” and “Starting a new chapter in a math book”; Hopko et al., 2003). Participants were asked to rate the level of anxiety associated with each situation on a 5-point Likert scale (maximum score is 45 points). Despite its brevity, it compares favorably with more extensive self-report measures of math anxiety such as the 98-item Math Anxiety Rating Scale with correlations reaching 0.85 (Ashcraft and Moore, 2009). The internal consistency of the scale was *α* = 0.90.

The Spielberger State-Trait Anxiety Inventory (STAI A-B; Spielberger et al., 1970) was administered to all participants during electrode preparation to measure situational and trait anxiety levels. The Greek version of this scale has adequate internal consistency (*α* = 0.92; Fountoulakis et al., 2006).

### Electroencephalographic Recordings

EEG recordings were performed in a dark and sound attenuated room during performance of each of the four arithmetic tasks and the three n-back conditions. Participants were seated in a comfortable chair and the stimuli were presented on a monitor located about 80 cm in front of the participant. ERPs, time-locked to the onset of each visual stimulus, were recorded via a Neurofax EEG-1200 system from 57 electrode sites according to a modified international 10/10 system using an Electrocap (Fp1, Fp2, F3, F4, C3, C4, P4, O1, O2, F7, F8, T7, T8, P7, P8, Fz, Cz, Pz, TP8, Afz, FCz, CPz, FC1, FC2, CP1, CP2, FC5, FC6, CP5, CP6, Fpz, Oz, F1, Poz, F2, C1, C2, P1, P2, AF3, AF4, FC3, FC4, CP3, CP4, PO3, PO4, F5, F6, C5, C6, P5, P6, FT7, FT8, TP7, referenced offline to linked mastoids). Vertical and horizontal eye movements were recorded through EOG from left/right canthal, supra- and infra-orbital electrodes. All electrode impedances were kept below 2 kΩ. High- and low-pass filters were set at 0.004 and 200 Hz, respectively, with a sampling rate of 500 Hz. Recorded epoch length was 1200 ms including a 200 ms prestimulus baseline. Prior to segmentation, signals were filtered offline between 0.5 and 45 Hz (with a notch filter at 47–53 Hz) and submitted to an ICA procedure (extended-ICA; Bell and Sejnowski, 1995) in order to identify components reflecting ocular artifacts, which were then filtered using the REGICA method (Klados et al., 2009, 2011) employing the algorithm proposed by Schlögl et al. (2007). Resulting waveforms were inspected visually and epochs containing visible artifacts in the first 500 ms post-stimulus were removed from further analyses (<10% of epochs).

Participants were asked to avoid alcohol intake on the day before and caffeine consumption on the day of the experiment; they were also asked to sleep as adequately and comfortably as possible on the night before. All recordings were performed in mid-morning sessions.

### Analyses

#### Anxiety and Task Performance

Response accuracy on the n-back and arithmetic tasks was assessed with d Prime (*d*^′^) which takes into account the proportion of hits (*H*) and false alarms (*F*) using the following formula:

d' = z(H) - z(F)

where *z* *(p), p* ∈ [0,1], is the inverse cumulative probability function of the normal distribution (Gale and Perkel, 2010). The reaction time (RT) of each trial was also recorded automatically by the stimuli presentation software (SuperLab^1^).

The effect of math anxiety on performance was assessed at the group level through three-way ANOVAs on *d*^′^ and RT with Math Anxiety Group and Gender as the between subjects variables and Arithmetic Task (Single Digit Addition, Single Digit Multiplication, Two-digit Addition, Two Digit Multiplication) or n-Back Condition (1-back, 2-back, 3-back) as the within subjects factor. Gender effects were explored given previous reports of higher self-reported math anxiety among women (e.g., Hopko et al., 2003). Self-reported, state and trait anxiety were used as covariates to ensure that math anxiety group effects were not affected by individual differences on levels of general anxiety.

#### ERP Amplitude

**Figure 1:**
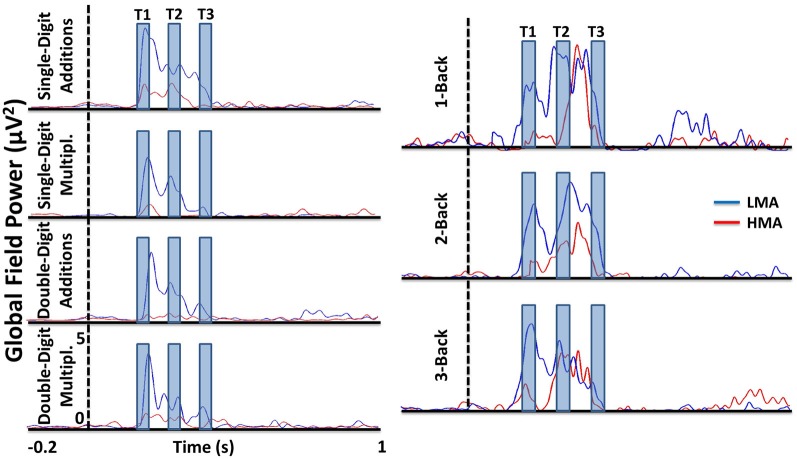
**Global field power (GFP) waveforms in group level for each task separately showing that there are three time windows mainly which is used in the amplitude analyses.** The GFP for each was computed for each task by considering only the cleaned trials.

Time windows used to compute ERP amplitude measures were determined upon inspection of global field power (GFP) waveforms separately for each group. As shown in Figure 1, time-locked activity was observed mainly during the first 450 ms post-stimulus with discernible peaks between 180–220 ms (T1), 280–320 ms (T2), and 380–420 ms (T3) in the n-back and arithmetic tasks. Negligible time-locked activity was detected after approximately 500 ms post-stimulus onset. Preliminary analyses were performed separately at each electrode, arithmetic task, n-back condition, and time window on mean ERP amplitude during T1, T2, and T3 time windows using ANOVAs with Math Anxiety Group (LMA, HMA) as the between subjects variable.

The False Discovery Rate (FDR; Benjamini and Hochberg, 1995) method was used to adapt the extracted *p*-values in order to control for type I error in an attempt to identify electrode sites where math anxiety effects might be present. Subsequently, three-way ANOVAs with Math Anxiety Group and Gender as the between subjects variables and Arithmetic Task (Single Digit Addition, Single Digit Multiplication, Two-digit Addition, Two Digit Multiplication) or n-Back Condition (1-back, 2-back, 3-back) as the within subjects factor were performed on electrode sites and time windows identified through the preliminary analyses in order to ascertain if math anxiety effects were moderated by arithmetic task or working memory load. Self-reported, state and trait anxiety were used as covariates in these analyses.

## Results

### Anxiety and Task Performance

**Figure 2:**
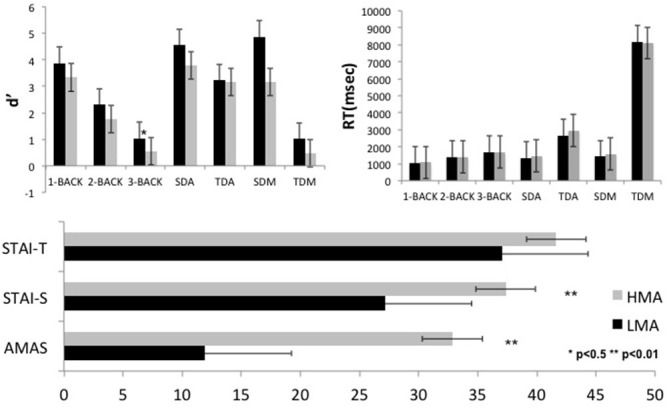
**Average performance indices (sensitivity [*d*^′^] and reaction time (RT)) of the low and high math anxiety groups (LMA, HMA, respectively) during performance of the arithmetic and working memory tasks are displayed in the upper panel.** Self-reported average math and general state and trait anxiety scores are shown in the lower panel. Error bars stand for the standard deviation, while statistical significance of group differences using *t*-tests is indicated by stars. Abbreviations; SDA, Single Digit Additions; TDA, Two Digit Additions; SDM, Single Digit Multiplications; TDM, Two Digit Multiplications.

In addition to AMAS scores on which the two groups differed by design, higher levels of situational anxiety were reported by participants in the HMA as compared to the LMA group, *t*_(30)_ = 3.94, *p* = 0.0001, yet the two groups showed comparable levels of trait anxiety (*p* > 0.10; see Figure 2 and Supplementary Table 1). With the exception of the expected main effect of gender on AMAS scores (*p* = 0.005, women > men), there were no other main effects or interactions involving gender. The Pearson correlation between AMAS and state anxiety was moderate (*r* = 0.575, *p* = 0.001) and much lower between AMAS and trait anxiety (*r* = 0.274, *p* = 0.129) in accordance with the original standardization study of AMAS (Hopko et al., 2003).

#### Arithmetic Tasks

As shown in Supplementary Table 1, after controlling for general state and trait anxiety levels, the tendency for higher RTs and lower *d*^′^ by high-math anxious participants did not reach significance on any task (*p* > 0.05).

Although the Group by Task interactions did not approach significance (*p* > 0.3), there were significant main effects of Task on both RT, *F*_(3, 90)_ = 117.14, *p* = 0.0001, and *d*^′^, *F*_(3, 90)_ = 117.23, *p* = 0.0001. Pairwise tests revealed comparable RT and *d*^′^ for the two single-digit tasks with significant drop in performance on two-digit addition and a further significant reduction on the two-digit multiplication task (both RT and *d*^′^; *p* = 0.0001 for all comparisons).

#### N-Back Task

Although the Group by Condition interaction did not approach significance (*p* > 0.7), planned pairwise comparisons revealed that the Group differences on accuracy were significant only during the 3-back condition. Working memory load however exerted the expected linear reduction in accuracy (main Condition effect: *F*_(2, 60)_ = 97.38, *p* = 0.0001) and corresponding increase in RT, *F*_(2, 60)_ = 37.70, *p* = 0.0001, with increasing memory load (*p* < 0.0001 for the linear trends, with *p* > 0.2 for the respective quadratic trends). Gender did not exert main or interaction effects on performance measures.

### ERP Amplitude

#### Arithmetic Tasks

**Figure 3:**
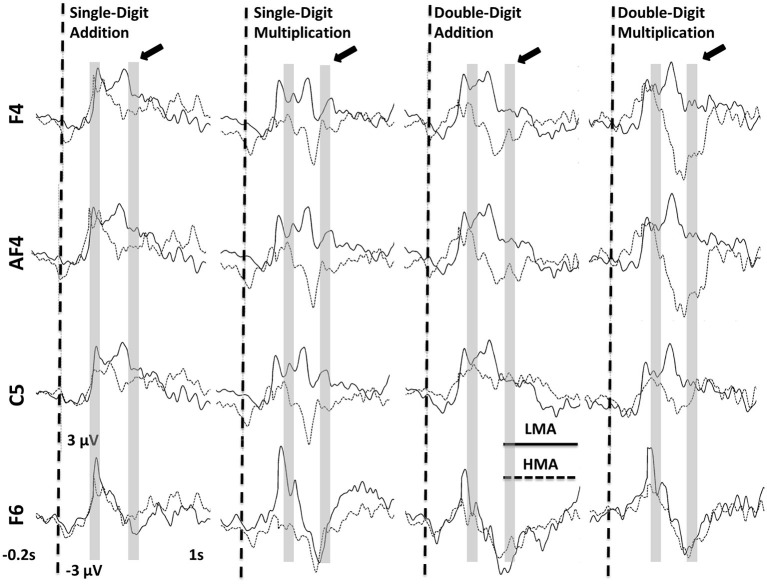
**Averaged ERPs recorded at frontocentral sites showing significant Math Anxiety Group main effects across arithmetic tasks at 180–220 (T1) and 380–420 ms (T3) (shaded regions).** The arrow points to the window where a significant math anxiety group effect was found during the n-back task (T3).

**Figure 4:**
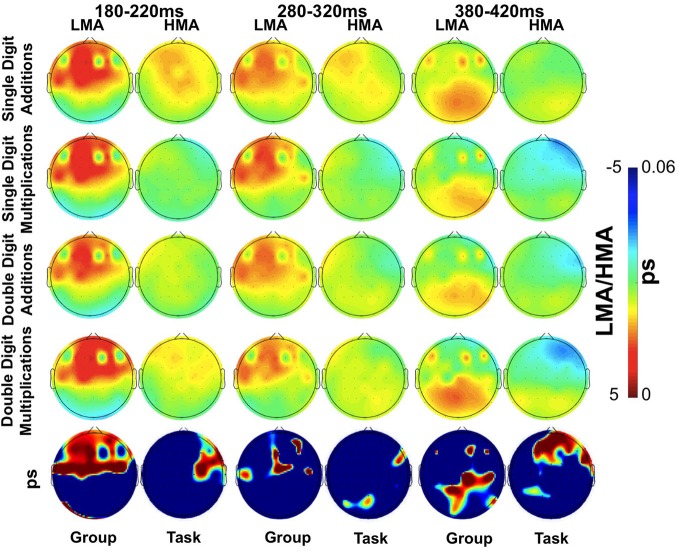
**Scalp distribution of mean ERP amplitudes at each of three time windows (180–220, 280–320, and 380–420 ms) during performance of the arithmetic tasks as a function of Math Anxiety group (LMA, HMA).** The distribution of False Discovery Rate (FDR)-corrected *p* values for the Group or Task main effect is shown in the lower row of images.

Significant Math Anxiety Group main effects (*p* < 0.002), indicating higher ERP amplitudes for the LMA than the HMA group, were found at the following frontocentral sites: F4, F2, AF4, FC4, F1, C1, and C5 between 180 and 220 ms (T1) and at Fz and F6 between 380 and 420 ms (T3). Corresponding waveforms at four representative sites are shown in Figure 3. Non-significant trends in the same direction were found at F4, FC2, AF4, C5, and C6 (0.003 > *p* > 0.01; T2) and at the latest time window (T3) at P8, Cz, Pz, CPz, C2, C6, and F5 (0.009 > *p* > 0.01). Voltage contour maps (Figure 4) illustrate the presence of a widely distributed positivity at frontocentral sites during early processing stages (across tasks), which shift to more posterior locations between 380 and 420 ms post-stimulus onset. Importantly this positivity was greatly reduced among participants who reported high levels of math anxiety. When controlling for individual differences in state and trait general anxiety task main effects on ERP amplitude did not reach significance.

#### N-Back Task

Math Anxiety Group main effects (LMA > HMA) meeting the stringent alpha level of 0.002 were restricted to three sites: FCz, C1, C5 (between 180–220 and 280–320 ms). Mean amplitude during 380–420 ms increased linearly with working memory load (1-back > 2-back > 3-back) at CP5 and CP3 (*p* < 0.002). There were no other main effects or interactions.

## Discussion

Electrophysiological data presented here support the hypothesis that higher levels of self-reported math anxiety are associated with lower cortical activation during performance of simple arithmetic calculation tasks, especially when the MA individuals didn’t achieve same performance as their control peers. An interesting finding that is discussed in more detail below, is that these effects were first evident during the early processing stages of task performance (i.e., within the first 200 ms after stimulus onset), several 100 ms before the participants’ responses were registered. With respect to the second goal of the study, Math Anxiety group effects were also documented on ERP amplitude during performance of the n-back task, albeit less extensively than the effects during performance of the arithmetic tasks. Moreover, the data corroborated our hypotheses that math anxiety group effects on electrophysiological measures were not significantly affected by individual differences in general, negative emotional reactivity (trait or state).

Despite earlier mixed results on the effects of anxiety on task-related ERP measures (e.g., Knyazev et al., 2004; Amodio et al., 2008; Aarts and Pourtois, 2010; Ansari and Derakshan, 2011; Judah et al., 2013; Suárez-Pellicioni et al., 2013), studies are generally more consistent in revealing reduced indices of cortical engagement with increased levels of anxiety. For instance, Fales et al. (2008) showed decreased sustained activity during a working memory task in several prefrontal and parietal regions participants reporting high levels of trait anxiety. Reduced prefrontal activity and slower RTs in a response conflict task have been reported by Bishop (2009), while Denkova et al. (2010) also reported anxiety-related reduction in hemodynamic activity in occipital and ventromedial prefrontal cortex.

The presented findings support that the significant differences between our two groups, are mainly located in the (pre-) frontal cortex. According to the existing literature (Smith and Jonides, 1997; Miyake et al., 2000; Fuster, 2002) the frontal cortex is mainly involved in working memory and more specifically in updating the working memory representations Smith and Jonides, 1997). Taking into account that MA strongly affects the working memory’s functioning, as well as our experimental setup, which is consisted by repeated trial that need item updating, the results can be interpreted accordingly. Another function that (pre-) frontal cortex is responsible for, is the temporal organization of actions towards a cognitive goal (Miyake et al., 2000). Considering that MA individuals are extremely reliant on the strategy they use, this pattern incommodes participants who are laboring under restricted working memory resources (Ashcraft and Ridley, 2005; Ashcraft and Krause, 2007).

From a neurocognitive standpoint, our findings are only partially consistent with the predictions of the Attentional Control Theory (ACT; Eysenck et al., 2007), which postulates that high levels of anxiety impair the ability to allocate cognitive resources to the task at hand and in turn affect cognitive efficiency (typically by reducing processing speed and prolonging RTs). Although a tendency for increased RTs and reduced response accuracy was evident for high math anxious participants, these effects did not reach significance when general trait and state anxiety scores were entered as covariates. Overall, comparable performance of the two math anxiety groups suggests that elevated trait math anxiety levels were not sufficient to significantly impair performance on simple calculation tasks. This conclusion appears to hold at least for the present sample of university students without history of learning disability. Specific math-related anxiety, on the other hand, was sufficient to alter neurophysiological processes reflected in the early portion of the ERP, which is often linked to attentional processes (Valdes-Sosa et al., 1998). This finding is consistent with the view that math anxiety interfered with cognitive resource allocation as postulated by ACT. The observation that math anxiety effects on ERPs were also evident during performance of an n-back task involving numeric stimuli is also consistent with ACT.

An alternative developmental/educational account posits that negative emotions regarding their adequacy in math develop “naturally” in students who are not as cognitively adept to acquire the most demanding math skills. Such emotional responses and cognitions are then likely to prevent them from engaging in academic activities related to math, therefore widening the gap with their more adept peers. Over the years these avoidance behaviors may further reinforce negative emotional reactions toward math tasks (Richardson and Suinn, 1972; Ashcraft and Ridley, 2005; Maloney and Beilock, 2012). In the sense that this account also predicts deficient engagement of a wide range of cognitive (and neural) processes in response to numeric stimuli in the context of arithmetic tasks, it may also be consistent with the present results.

It should be noted that the design of the experiment and analyses employed in the current study did not permit us to assess additional predictions of ACT, namely that anxious individuals are also likely to exert greater cognitive effort and make use of more cognitive resources in order to achieve performance standards displayed by persons with low anxiety. Such compensatory cognitive strategies are likely to engage neurophysiological processes which will take place during later stages of numeric stimulus processing, extending beyond the narrow time window examined here. Moreover, both the precise timing and type of compensatory strategies engaged by high math-anxious participants are likely to show significant individual variability which will further reduce their capacity to produce time-locked ERPs. This may further account for the failure to find clear evidence of ERP modulation as a function of task difficulty (for arithmetic tasks) and working memory load (for the n-back tasks).

The limited sensitivity of the method used to measure and model cortical activation in the present study should be taken into account in interpreting the current results. Thus, both the magnitude of cortical activation were assessed at the sensor level, not possessing adequate spatial resolution to detect compensatory increases in neural responsivity as predicted by ACT. fMRI and EEG source-level data derived in the context of similar experimental paradigms are forthcoming to address this issue (Babiloni et al., 2004).

Additional limitations inherent to the study design should be noted. Thus, failure to find MA effects in later portions of the ERPs may have been an artifact of the time window used to estimate the aformentioned parameters, so that critical operations involved in the more demanding two-digit calculation tasks may have taken place at later time windows (which were not reliably time-locked and estimated in the present study). Failure to find significant variability in GFP after approximately 500 ms post-stimulus onset may simply reflect difficulty in eliciting time-locked neurophysiological activity in response to tasks that require extensive processing. This limitation may also be manifested in the absence of clear-cut arithmetic task main effects and interactions, given that the analysis window empirically established on the basis of GFP waveforms captured neurophysiological activity elicited during the early stages of arithmetic calculation. These stages are likely to be dominated by processes common to the elaboration of numeric stimuli across tasks. Moreover, future studies should attempt to manipulate arithmetic task difficulty more systematically and perhaps also employ measures of synchronization/desynchronization at the single trial level in order to circumvent the stationarity requirement in the analysis of average ERP data.

A final important limitation of the present study, relates to the sample size which may have been sufficient for group-level analyses as well as for preliminary estimation of bivariate associations. However, a larger study size is required to perform more complex analyses, such as mediated regression, which will be ideally suited to assess the hypothesized role of ERPs’ parameters as mediators of the association between math anxiety and performance. Larger data sets are also required in order to assess the potential non-linear effects of math anxiety and the interaction of affective and cognitive abilities. Generally, however, it has been difficult to model the complex associations between math anxiety and corresponding beliefs and schemas regarding mathematics, cognitive capacities (such as processing speed, working memory, and problem solving ability), and actual performance on arithmetic tasks that vary in difficulty. One reason for this difficulty may be that associations may not be linear—it is well known that the relation between anxiety and performance is curvilinear and the shape of the anxiety-performance function may change with task difficulty. This quest is further complicated by the interdependence of performance measures as indices of math capacity. Thus, anxiety may have a positive effect on RT and calculation accuracy up to a certain level beyond which reductions in RT may be associated with reduced accuracy (Ashcraft and Faust, 1994; Beilock et al., 2007). Other individuals may instead be more cautious at relatively low levels of MA leading to increased RTs and accuracy. As suggested by Berggren et al. (2013) it is important to establish putative interactions between cognitive load, processing efficiency and effectiveness by manipulating participant motivation (both within and between subjects) as well as other relatively stable personality traits may moderate the effects of situational anxiety in EEG/ERP parameters (see for instance Reiser et al., 2012).

Despite the aforementioned limitations, this is the first study investigating electrophysiological measures of cortical function during the solution of arithmetic tasks varying in difficulty and operational complexity as a function of self-reported math anxiety. Our findings indicate that higher levels of self-reported math anxiety are associated with lower cortical activation during the early stages of performance of simple arithmetic calculation tasks. Regardless of the precise neurocognitive mechanisms, an important implication of the present results concerns the need to take into account individual differences in math anxiety levels in neuroimaging studies involving numerical stimuli.

## Conflict of Interest Statement

The authors declare that the research was conducted in the absence of any commercial or financial relationships that could be construed as a potential conflict of interest.

## Footnotes

^1^http://www.superlab.com/

## References

[B1] Aarts K. Pourtois G. (2010). Anxiety not only increases, but also alters early error-monitoring functions. Cogn. Affect. Behav. Neurosci. 10, 479–492. 10.3758/cabn.10.4.479 21098809

[B2] Amodio D. M. Devine P. G. Harmon-Jones E. (2008). Individual differences in the regulation of intergroup bias: the role of conflict monitoring and neural signals for control. J. Pers. Soc. Psychol. 94, 60–74. 10.1037/0022-3514.94.1.60 18179318

[B3] Ansari T. L. Derakshan N. (2011). The neural correlates of cognitive effort in anxiety: effects on processing efficiency. Biol. Psychol. 86, 337–348. 10.1016/j.biopsycho.2010.12.013 21277934

[B4] Ashcraft M. H. Faust M. W. (1994). Mathematics anxiety and mental arithmetic performance: an exploratory investigation. Cogn. Emot. 8, 97–125. 10.1080/02699939408408931

[B49] Ashcraft M. H. Krause J. A. (2007). Working memory, math performance, and math anxiety. Psychon. Bull. Rev. 14, 243–248. 10.3758/BF03194059 17694908

[B5] Ashcraft M. H. Moore A. M. (2009). Mathematics anxiety and the affective drop in performance. J. Psychoed. Assess. 27, 197–205. 10.1177/0734282908330580

[B6] Ashcraft M. H. Ridley K. S. (2005). “Math anxiety and its cognitive consequences,” in Handbook of Mathematical Cognition, ed. Campbell J. I. D. (New York, NY: Psychology Press), 315–327.

[B7] Ashcraft M. H. (2002). Math anxiety: personal, educational and cognitive consequences. Curr. Dir. Psychol. Sci. 11, 181–185. 10.1111/1467-8721.00196

[B8] Babiloni C. Binetti G. Cassetta E. Cerboneschi D. Dal Forno G. Del Percio C.. (2004). Mapping distributed sources of cortical rhythms in mild alzheimer’s disease. A multicentric EEG study. Neuroimage 22, 57–67. 10.1016/j.neuroimage.2003.09.028 15109997

[B9] Basten U. Stelzel C. Fiebach C. J. (2012). Trait anxiety and the neural efficiency of manipulation in working memory. Cogn. Affect. Behav. Neurosci. 12, 571–588. 10.3758/s13415-012-0100-3 22644759 PMC3400031

[B10] Beilock S. L. Rydell R. J. McConnell A. R. (2007). Stereotype threat and working memory: mechanisms, alleviation and spillover. J. Exper. Psychol. Gen. 136, 256–276. 10.1037/e633982013-073 17500650

[B11] Bell A. J. Sejnowski T. J. (1995). An information-maximization approach to blind separation and blind deconvolution. Neural Comput. 7, 1129–1159. 10.1162/neco.1995.7.6.1129 7584893

[B12] Benjamini Y. Hochberg Y. (1995). Controlling the false discovery rate: a practical and powerful approach to multiple testing. J. R. Stat. Soc. Ser. B 57, 289–300.

[B13] Berggren N. Richards A. Taylor J. Derakshan N. (2013). Affective attention under cognitive load: reduced emotional biases but emergent anxiety-related costs to inhibitory control. Front. Hum. Neurosci. 7:188. 10.3389/fnhum.2013.00188 23717273 PMC3652291

[B14] Betz N. E. (1978). Prevalence, distribution and correlates of math anxiety in college students. J. Couns. Psychol. 25, 441–448. 10.1037/0022-0167.25.5.441

[B15] Bishop S. J. (2009). Trait anxiety and impoverished prefrontal control of attention. Nat. Neurosci. 12, 92–98. 10.1038/nn.2242 19079249

[B17] Denkova E. Wong G. Dolcos S. Sung K. Wang L. Coupland N.. (2010). The impact of anxiety-inducing distraction on cognitive performance: a combined brain imaging and personality investigation. PLoS One 5:e14150. 10.1371/journal.pone.0014150 21152391 PMC2994755

[B18] Eysenck M. W. Derakshan N. Santos R. Calvo M. G. (2007). Anxiety and cognitive performance: attentional control theory. Emotion 7, 336–353. 10.1037/1528-3542.7.2.336 17516812

[B19] Fales C. L. Barch D. M. Burgess G. C. Schaefer A. Mennin D. S. Gray J. R.. (2008). Anxiety and cognitive efficiency: differential modulation of transient and sustained neural activity during a working memory task. Cogn. Affect. Behav. Neurosci. 8, 239–253. 10.3758/cabn.8.3.239 18814461

[B20] Fennema E. (1989). “The study of affect and mathematics: a proposed generic model for research,” in Affect and Mathematical Problem Solving, eds McLeod D. Adams V. (New York: Springer), 205–219.

[B21] Fountoulakis K. N. Papadopoulou M. Kleanthous S. Papadopoulou A. Bizeli V. Nimatoudis I.. (2006). Reliability and psychometric properties of the greek translation of the state-trait anxiety inventory form Y: preliminary data. Ann. Gen. Psychiatry 5:2. 10.1186/1744-859X-5-2 16448554 PMC1373628

[B48] Fuster J. M. (2002). Frontal lobe and cognitive development. J. Neurocytol. 31, 373–385. 10.1023/A:1024190429920 12815254

[B22] Gale S. D. Perkel D. J. (2010). A basal ganglia pathway drives selective auditory responses in songbird dopaminergic neurons via disinhibition. J. Neurosci. 30, 1027–1037. 10.1523/JNEUROSCI.3585-09.2010 20089911 PMC2824341

[B23] Hopko D.R. Mahadevan R. Bare R. L. Hunt M. K. (2003). The Abbreviated Math Anxiety Scale (AMAS): construction, validity and reliability. Assessment 10, 178–182. 10.1177/1073191103010002008 12801189

[B24] Imbo I. Vandierendonck A. (2008). Effects of problem size, operation and working-memory span on simple-arithmetic strategies: differences between children and adults? Psychol. Res. 72, 331–346. 10.1007/s00426-007-0112-8 17457605

[B25] Jones W. J. Childers T. L. Jiang Y. (2012). The shopping brain: math anxiety modulates brain responses to buying decisions. Biol. Psychol. 89, 201–213. 10.1016/j.biopsycho.2011.10.011 22027087

[B26] Judah M. R. Grant D. M. Lechner W. V. Mills A. C. (2013). Working memory load moderates late attentional bias in social anxiety. Cogn. Emot. 27, 502–511. 10.1080/02699931.2012.719490 22963405

[B27] Klados M. A. Kanatsouli K. Antoniou I. Babiloni F. Tsirka V. Bamidis P. D.. (2013). A graph theoretical approach to study the organization of the cortical networks during different mathematical tasks. PLoS One 8:e71800. 10.1371/journal.pone.0071800 23990992 PMC3747176

[B29] Klados M. A. Papadelis C. L. Bamidis P. D. (2009). “REG-ICA: a new hybrid method for eog artifact rejection,” in 9th International Conference on Information Technology and Applications in Biomedicine. IEEE (Larnaca), 1–4.

[B28] Klados M. A. Papadelis C. Braunb C. Bamidisa P. D. (2011). REG-ICA: A hybrid methodology combining blind source separation and regression techniques for the rejection of ocular artifacts. Biomed. Signal Process. Control 6, 291–300. 10.1016/j.bspc.2011.02.001

[B45] Knyazev G. G. Savostyanov A. N. Levin E. A. (2004). Alpha oscillations as a correlate of trait anxiety. Int. J. Psychophysiol. 53, 147–160. 10.1016/j.ijpsycho.2004.03.001 15210292

[B31] LeFevre J. A. DeStefano D. Coleman B. Shanahan T. (2005). “Mathematical cognition and working memory,” in Handbook of Mathematical Cognition, ed. Campbell J. I. D. (New York, NY: Psychology Press Ltd.), 361–378.

[B32] Lyons I. M. Beilock S. L. (2012a). Mathematics anxiety: separating the math from the anxiety. Cereb. Cortex 22, 2102–2110. 10.1093/cercor/bhr289 22016480

[B33] Lyons I. M. Beilock S. L. (2012b). When math hurts: math anxiety predicts pain network activation in anticipation of doing math. PLoS One 7:e48076. 10.1371/journal.pone.0048076 23118929 PMC3485285

[B34] Ma X. (1999). A meta-analysis of the relationship between anxiety toward mathematics and achievement in mathematics. J. Res. Math. Educ. 30, 520–540. 10.2307/749772

[B35] Maloney E. A. Beilock S. L. (2012). Math anxiety: who has it, why it develops and how to guard against it. Trends Cogn. Sci. 16, 404–406. 10.1016/j.tics.2012.06.008 22784928

[B47] Miyake A. Friedman N. P. Emerson M. J. Witzki A. H. Howerter A. Wager T. D. (2000). The unity and diversity of executive functions and their contributions to complex “Frontal Lobe” tasks: a latent variable analysis. Cogn. Psychol. 41, 49–100. 10.1006/cogp.1999.0734 10945922

[B37] Qi S. Zeng Q. Luo Y. Duan H. Ding C. Hu W.. (2014). Impact of working memory load on cognitive control in trait anxiety: an ERP study. PLoS One 9:e111791. 10.1371/journal.pone.0111791 25369121 PMC4219777

[B38] Raghubar K. P. Barnes M. A. Hecht S. A. (2010). Working memory and mathematics: A review of developmental, individual difference and cognitive approaches. Learn. Indiv. Differ. 20, 110–122. 10.1016/j.lindif.2009.10.005

[B39] Reiser E. M. Schulter G. Weiss E. M. Fink A. Rominger C. Papousek I. (2012). Decrease of prefrontal-posterior EEG coherence: loose control during social-emotional stimulation. Brain Cogn. 80, 144–154. 10.1016/j.bandc.2012.06.001 22750775

[B40] Richardson F. C. Suinn R. M. (1972). The mathematics anxiety rating scale: psychometric data. J. Couns. Psychol. 19, 551–554. 10.1037/h0033456

[B41] Schlögl A. Keinrath C. Zimmermann D. Scherer R. Leeb R. Pfurtscheller G. (2007). A fully automated correction method of EOG artifacts in EEG recordings. Clin. Neurophysiol. 118, 98–104. 10.1016/j.clinph.2006.09.003 17088100

[B46] Smith E. E. Jonides J. (1997). Working memory: a view from neuroimaging. Cogn. Psychol. 33, 5–42. 10.1006/cogp.1997.0658 9212720

[B42] Spielberger C. Gorsuch R. Lushene R. (1970). Manual for the state-trait anxiety inventory. J. Educ. Psychol. 61, 386-391. 5474298

[B43] Suárez-Pellicioni M. Núñez-Peña M. Colomé A. (2013). Abnormal error monitoring in math-anxious individuals: evidence from error-related brain potentials. PLoS One 8:e81143. 10.1371/journal.pone.0081143 24236212 PMC3827466

[B44] Valdes-Sosa M. Cobo A. Pinilla T. (1998). Transparent motion and object-based attention. Cognition 66, B13–B23. 10.1016/s0010-0277(98)00012-2 9677765

