# Supplementary material for: ERP measures of math anxiety: how math anxiety affects working memory and mental calculation tasks?
Source: Front Behav Neurosci. 2015 Oct 26;9:282. doi: 10.3389/fnbeh.2015.00282 (PMC4620156; doi:10.3389/fnbeh.2015.00282)
Supplement: Supplementary file 1 [file Table_1.docx]

Supplementary Material

Neural Correlates Of Math Anxiety: ERP And Functional Connectivity Measures

**Manousos A. Klados1,2*, Panagiotis Simos3,*, Sifis Micheloyannis4, Daniel Margulies1 and Panagiotis D. Bamidis2* Correspondence:**

Corresponding Author: klados@cbs.mpg.de

## Supplementary Tables

Table 1 – Anxiety scores and task performance for the two math anxiety groups.

|  | LMA (n=16) | | HMA (n=16) | |  |
| --- | --- | --- | --- | --- | --- |
|  | Mean | SD | Mean | SD | p* |
| AMAS | 11.94 | 1.69 | 32.87 | 3.96 | **.0001** |
| STAI-State | 27.18 | 4.94 | 37.37 | 9.2 | **.0001** |
| STAI-Trait | 37.06 | 6.72 | 41.63 | 8.5 | .10 |
| RT | | | | | |
| 1-back | 1125.12 | 396.31 | 2049.58 | 428.85 | **.0001** |
| 2-back | 1819.03 | 705.27 | 2640.22 | 729.05 | **.003** |
| 3-back | 2320.78 | 1040.87 | 3121.62 | 1349.53 | .07 |
| Single-Digit Addition | 1577.24 | 417.32 | 2599.76 | 555.96 | **.0001** |
| Two-Digit Addition | 4094.61 | 1315.21 | 5479.57 | 1364.26 | **.007** |
| Single-Digit Multiplication | 1661.49 | 469.24 | 2757.03 | 780.82 | **.0001** |
| Two-Digit Multiplication | 15098.43 | 7599.91 | 15736.32 | 6361.43 | .79 |
| $d'$ | | | | | |
| 1-back | 3.88 | 1.32 | 3.33 | 1.43 | .27 |
| 2-back | 2.32 | 0.57 | 1.77 | 0.85 | **.04** |
| 3-back | 1.04 | 0.55 | 0.56 | 0.45 | **.01** |
| Single-Digit Addition | 4.55 | 1.17 | 3.78 | 0.98 | **.05** |
| Two-Digit Addition | 3.22 | 1.00 | 3.17 | 1.58 | .06 |
| Single-Digit Multiplication | 4.87 | 1.02 | 3.17 | 1.58 | .89 |
| Two-Digit Multiplication | 1.02 | 0.80 | 0.47 | 0.71 | **.05** |

*t-tests comparing the two groups. LMA: Low Math Anxiety, HMA: High Math Anxiety.
