# Supplementary figures and images for: ERP measures of math anxiety: how math anxiety affects working memory and mental calculation tasks?
Source: Front Behav Neurosci. 2015 Oct 26;9:282. doi: 10.3389/fnbeh.2015.00282 (PMC4620156; doi:10.3389/fnbeh.2015.00282)

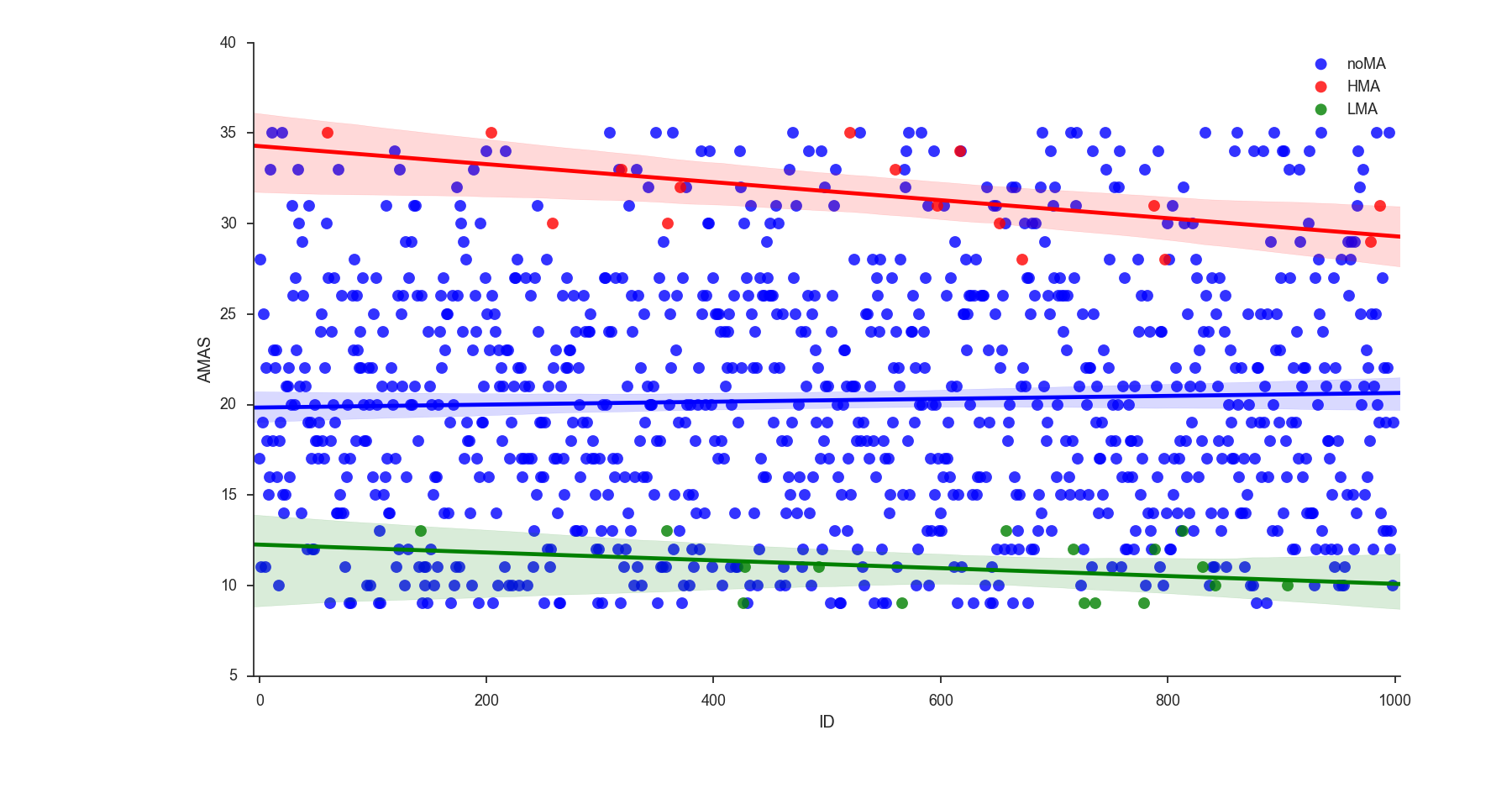

Supplement: Supplementary file 2 [file Image_1.png]
